# New semisynthetic vinca alkaloids: chemical, biochemical and cellular studies

**DOI:** 10.1038/sj.bjc.6690218

**Published:** 1999-03

**Authors:** F Orosz, B Comin, B Raïs, J Puigjaner, J Kovács, G Tárkányi, T Ács, T Keve, M Cascante, J Ovádi

**Affiliations:** Institute of Enzymology, Biological Research Center, Hungarian Academy of Sciences, POB 7, H-1518, Hungary; Department of Biochemistry and Molecular Biology, Faculty of Chemistry, Barcelona, Spain; Department of General Zoology, Faculty of Sciences, University of Eötvös Loránd, POB 330, H-1445, Hungary; Chemical Works of Gedeon Richter Ltd, Chemical Works of Gedeon Richter Ltd, Budapest, POB 27, H-1475, Hungary

**Keywords:** vinca alaloid, bis-indol, vinblastine, vincristine, microtubule, calmodulin, antagonism

## Abstract

A new semisynthetic anti-tumour bis-indol compound, KAR-2 [3′-(β-chloroethyl)-2′,4′-dioxo-3,5′-spiro-oxazolidino-4-deacetoxy-vinblastine] with lower toxicity than vinca alkaloids used in chemotherapy binds to calmodulin but, in contrast to vinblastine, does not exhibit anti-calmodulin activity. To investigate whether the modest chemical modification of bis-indol structure is responsible for the lack of anti-calmodulin potency and for the different pharmacological effects, new derivatives have been synthesized for comparative studies. The synthesis of the KAR derivatives are presented. The comparative studies showed that the spiro-oxazolidino ring and the substitution of a formyl group to a methyl one were responsible for the lack of anti-calmodulin activities. The new derivatives, similar to the mother compounds, inhibited the tubulin assembly in polymerization tests in vitro, however their inhibitory effect was highly dependent on the organization state of microtubules; bundled microtubules appeared to be resistant against the drugs. The maximal cytotoxic activities of KAR derivatives in in vivo mice hosting leukaemia P388 or Ehrlich ascites tumour cells appeared similar to that of vinblastine or vincristine, however significant prolongation of life span could be reached with KAR derivatives only after the administration of a single dose. These studies plus data obtained using a cultured human neuroblastoma cell line showed that KAR compounds displayed their cytotoxic activities at significantly higher concentrations than the mother compounds, although their antimicrotubular activities were similar in vitro. These data suggest that vinblastine/vincristine damage additional crucial cell functions, one of which could be related to calmodulin-mediated processes. © 1999 Cancer Research Campaign


British Journal of Cancer (1999) 79(9/10), 1356–1365
© 1999 Cancer Research Campaign
Article no. bjoc.1998.0218

### New semisynthetic vinca alkaloids: chemical, biochemical and cellular studies

**F Orosz[1], B Comin[2], B Raïs[2], J Puigjaner[2], J Kovács[3], G Tárkányi[4], T Ács[4], T Keve[4], M Cascante[2] and J Ovádi[1]**

[1]Institute of Enzymology, Biological Research Center, Hungarian Academy of Sciences, Budapest, H-1518, POB 7, Hungary; [2]Department of Biochemistry and Molecular Biology, Faculty of Chemistry, University of Barcelona, Barcelona, Catalonia, Spain; [3]Department of General Zoology, Faculty of Sciences, University of Eötvös Loránd, Budapest, H-1445, POB 330, Hungary; [4]Chemical Works of Gedeon Richter Ltd, Budapest, H-1475, POB 27, Hungary

**Summary** A new semisynthetic anti-tumour bis-indol compound, KAR-2 [3′-(β-chloroethyl)-2′,4′-dioxo-3,5′-spiro-oxazolidino-4-deacetoxy-vinblastine] with lower toxicity than vinca alkaloids used in chemotherapy binds to calmodulin but, in contrast to vinblastine, does not exhibit anti-calmodulin activity. To investigate whether the modest chemical modification of bis-indol structure is responsible for the lack of anti-calmodulin potency and for the different pharmacological effects, new derivatives have been synthesized for comparative studies. The synthesis of the KAR derivatives are presented. The comparative studies showed that the spiro-oxazolidino ring and the substitution of a formyl group to a methyl one were responsible for the lack of anti-calmodulin activities. The new derivatives, similar to the mother compounds, inhibited the tubulin assembly in polymerization tests in vitro, however their inhibitory effect was highly dependent on the organization state of microtubules; bundled microtubules appeared to be resistant against the drugs. The maximal cytotoxic activities of KAR derivatives in in vivo mice hosting leukaemia P388 or Ehrlich ascites tumour cells appeared similar to that of vinblastine or vincristine, however significant prolongation of life span could be reached with KAR derivatives only after the administration of a single dose. These studies plus data obtained using a cultured human neuroblastoma cell line showed that KAR compounds displayed their cytotoxic activities at significantly higher concentrations than the mother compounds, although their antimicrotubular activities were similar in vitro. These data suggest that vinblastine/vincristine damage additional crucial cell functions, one of which could be related to calmodulin-mediated processes.

**Keywords:** vinca alkaloid; bis-indol; vinblastine; vincristine; microtubule; calmodulin antagonism

The numerous antimitotic drugs already developed and therapeutically used for a large number of pathologies belong to different classes based on their chemical structure and other distinctive features. Antimitotic drugs usually target the tubulin/microtubule network of the cytoskeleton that is formed by assembly of cytoplasmic tubulin dimers. The stability of the different microtubular structures varies considerably; some of their functions depend on their lability. The lability is augmented by the extreme sensitivity to various drugs. Several synthetic and natural compounds interact specifically with tubulin and/or microtubule, fundamentally destroying its dynamic character and leading to cell death. A large number of these agents are plant derived (Lin et al, 1988).

Vinca alkaloids, vinblastine and vincristine are potent antimitotic agents, they have been effectively used in the cancer therapy (Dustin, 1984). Because of their extensive clinical use, it is important to synthesize additional agents having at least the same biological activity. Numerous semisynthetic derivatives of vinca alkaloids have been synthesized (Eli Lilly, Indianapolis, IN, USA), however only few data for tubulin binding are available. The chemical conversion of indols occurring in relatively large amounts in the plant extract into potent semisynthetic anti-tumour agents is also motivated by, on one hand, the extensive need for potent anti-tumour agents in clinical chemotherapy and, on the other hand, by the fact that the known drugs, vinblastine and vincristine, have undesired side-effects.

Although it has been suggested that the primary target of the bis-indol alkaloids is the microtubular network, they also bind to calmodulin, a ubiquitous $Ca^{2+}$ receptor protein that is involved in the cell proliferation (De Brabander et al, 1980) and a number of other physiological processes. Recently, we demonstrated that the bis-indol vinca alkaloids display significant anti-calmodulin activity (Molnár et al, 1995). The affinity of these drugs to tubulin (Liliom et al, 1995) and calmodulin (Molnár et al, 1995) in in vitro binding tests were found to be comparable. Nevertheless, it is unclear yet how the anti-calmodulin activity of these drugs is related to their desired and undesired pharmacological effects.

Recently, we reported (Orosz et al, 1997*a*,*b*) that a new bis-indol derivative, KAR-2 [3′-(β-chloroethyl)-2′,4′-dioxo-3,5′-spiro-oxazolidino-4-deacetoxy-vinblastine], with antimitotic activity interacts with tubulin as well as with calmodulin in in vitro systems. Electron microscopic studies on Chinese hamster ovary cell lines revealed that KAR-2 targets microtubular network destroying the cytoplasmic microtubular system. KAR-2 was found to be a powerful anti-tumour agent on mouse leukaemia P388 in vivo tests, however its administration did not induce neurotoxic side-effects (e.g. paralysis of bladder or lower extremities) as observed in the case of bis-indols routinely used in therapy. Moreover, we found that it was effective even in a single high dose, whereas vincristine using the same administration schedule was toxic or less effective than KAR-2 (Orosz et al, 1997*a*).

*Received 7 April 1998*
*Revised 25 June 1998*
*Accepted 4 August 1998*

*Correspondence to:* J Ovádi

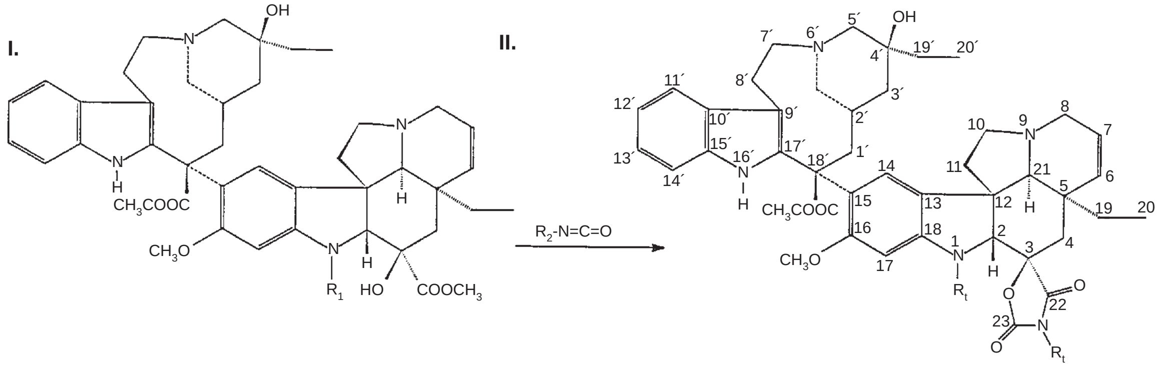

**Figure 1** Scheme of synthesis of KAR derivatives

| Compound (I) | Compound (II) | $R_1$ | $R_2$ |
|---|---|---|---|
| 4-Deacetoxy-vinblastine | KAR-2 | $CH_3$ | $CH_2CH_2Cl$ |
| 4-Deacetoxy-vincristine | KAR-3 | CHO | $CH_2CH_2Cl$ |
| 4-Deacetoxy-vinblastine | KAR-4 | $CH_3$ | $CH_2CH{=}CH_2$ |

KAR-2 was found to bind to calmodulin, however, in contrast to other biologically active bis-indols used in cancer chemotherapy, it did not show anti-calmodulin activity. Therefore, the binding does not necessarily cause anti-calmodulin activity, in spite of the fact that the binding affinities of the bis-indol derivatives examined to calmodulin are comparable. It was suggested that this unique character of KAR-2 could be one of the reasons of its low toxicity (Orosz et al, 1997*a,b*). To investigate whether the lack of anti-calmodulin potency resides only on the spiro-oxazolidino portion of KAR-2 and the modest chemical modification of bis-indol structure is responsible also for the different pharmacological effects, new derivatives have been synthesized for comparative studies.

In this paper, the synthesis and structural elucidation of KAR-2 and two further new bis-indol derivatives, KAR-3 and KAR-4 (Figure 1), is described which are analogues of vincristine and vinblastine respectively. These compounds were found to be powerful anti-tumour agents on mouse leukaemia P388 and Ehrlich ascites carcinoma in vivo tests and on neuroblastoma cell lines. Biochemical studies showed that they effectively impeded tubulin polymerization and specifically damaged single microtubules but not the bundled ones. KAR-3 displayed significantly higher anti-calmodulin and slightly higher anti-tubulin activity than the other two KAR derivatives.

### MATERIALS AND METHODS

### General methods

#### *Chemicals*

Indol alkaloids vinblastine and vincristine were isolated from *Catharanthus roseus* by the standard procedure carried out by Chemical Works of Gedeon Richter, Hungary. 4-Deacetoxy-vinblastine was isolated from *Catharanthus roseus* extract according to De Bruyn et al (1982). 4-Deacetoxy-vincristine was produced in acetone-methylene chloride from 4-deacetoxy-vinblastine using *n*-$Bu_4NMnO_4$ as oxidizing agent. Taxol, GTP and trifluoperazine were purchased from Sigma. All organic reagents for synthesis of the new compounds were obtained from Aldrich and were of the highest grade. All other chemicals were reagent grade commercial preparations.

#### *Proteins*

Tubulin was prepared from calf brain using the Weisenberg method modified by Na and Timasheff (1986). Purified tubulin was virtually free from microtubule-associated protein bands when run on overloaded sodium dodecyl sulphate polyacrylamide gel electrophoresis (SDS-PAGE). Tubulin was stored in 1 M sucrose, 10 mM sodium phosphate buffer, 0.5 mM magnesium chloride and 0.1 mM GTP, pH 7.0, at –80°C and dialysed against the appropriate buffer before use.

Calmodulin from bovine brain was purified to homogeneity using Phenyl-Sepharose (Pharmacia) chromatography (Gopalakrishna and Anderson, 1982). Its activity was checked by the phosphodiesterase test (Solti et al, 1983), or by phosphofructokinase [ATP: D-fructose 6-phosphate 1-phospho-transferase, EC 2.7.1.11] (PFK) test (Orosz et al, 1988).

PFK, aldolase and glycerol-3-phosphate dehydrogenase-triosephosphate isomerase purified from rabbit skeletal muscle were purchased in ammonium sulphate suspensions from Sigma or Boehringer. The enzyme suspensions were centrifuged at 10 000***g*** for 5 min. The pellets were suspended in the appropriate buffer, then the enzymes were gel filtered or dialysed in the same buffer to remove ammonium sulphate.

Protein concentrations were determined spectrophotometrically, using molar absorption coefficients of $1.06 \times 10^5$ $M^{-1}$ $cm^{-1}$ at 280 nm for tubulin (Na and Timasheff, 1986), $3.24 \times 10^3$ $M^{-1}$ $cm^{-1}$ at 276 nm for calmodulin (Watterson et al, 1976), $8.88 \times 10^4$ $M^{-1}$ $cm^{-1}$ at 280 nm for PFK (Hesterberg and Lee, 1982). Spectroscopic

© Cancer Research Campaign 1999

measurements were made on HP 8451A or JASCO V-550 spectrophotometers. Protein purity was determined by SDS-PAGE, as described by Laemmli (1970)

### Synthetic chemistry

### *Synthesis of KAR-2*

[3′-(β-chloroethyl)-2′,4′-dioxo-3,5′-spiro-oxazolidino-4-deacetoxy-vinblastine] (Figure 1B; $R_1$, $CH_3$; $R_2$, $CH_2CH_2Cl$). Ten grams (13.28 mmol) 4-deacetoxy-vinblastine (Figure 1A; $R_1$, $CH_3$) was dissolved in 100 ml absolute tetrahydrofurane followed by the addition of 8.0 ml (10.1 g; 95.9 mmol) chloroethyl-isocyanate. After 2 h incubation at room temperature, 200 ml dried toluene was added to the reaction mixture. Then the mixture was kept at 80°C for 18 h, cooled to 2°C and 40 ml water and 2 ml 25% ammonia was added. The mixture was stirred for 30 min. After separation of the organic phase, it was washed with water (3 × 30 ml) and dried with potassium sulphate and evaporated on a water bath of 35–40°C at 20–27 hPa. The resulting material was chromatographed on silica gel (500 g, bead size of 0.040–0.063 mm) column (30 mm × 500 mm) using ethyl acetate/ethanol (9:1) mixture as eluent. The yield was 3.29 g (30%).

KAR-2: thin layer chromatography: $R_f$= 0.70–0.75 (9:1 ethyl acetate/ethanol); IR $\upsilon_{max}$ (potassium bromide): 3465, 1814, 1743, 1617, 1408, 1241, 1039, 741 $cm^{-1}$; MS-FAB *m/z* 826 $[M + H]^+$.

Sulphate salt: 3 g (3.63 mmol) pure base was dissolved in 5 ml ethanol to which 7 ml ethanol containing 0.363 g (3.63 mmol) sulphuric acid was added. The mixture was kept for 4 h at room temperature and then evaporated on a water bath at 40°C under a vacuum of 80 hPa to a final volume of 5–6 ml. The salt was precipitated from the mixture by adding 20 ml ether then filtered and dried at maximum 30°C at 13 hPa for 8–15 h, resulting in a white, crystalline powder. The yield was 3.05 g (83%); m.p. > 300°C (decomposed). Optical rotation (1% solution in water): $[\alpha_D]_0^{25}$ = +3.3°; $[\alpha_D]^{25}_{Hg\ 578}$ = +3.6°; $[\alpha_D]^{25}_{Hg\ 546}$ = +4.5°; $[\alpha_D]^{25}_{Hg\ 436}$ = +12.9°; $[\alpha_D]^{25}_{Hg\ 365}$ = +36.7°.

### *Synthesis of KAR-3*

[3′-(β-chloroethyl)-2′,4′-dioxo-3,5′-spiro-oxazolidino-4-deacetoxy-vincristine] (Figure 1B, $R_1$, CHO; $R_2$, $CH_2CH_2Cl$). The steps of synthesis of KAR-3 are the same as that of KAR-2, but starting from the required (10 g, 11.9 mmol) deacetoxy-vincristine (Figure 1A; $R_1$, CHO). The yield was 29% and 86% for KAR-3 and KAR-3 sulphate respectively.

KAR-3: IR $\upsilon_{max}$ (potassium bromide): 3445, 1817, 1744, 1617, 1595, 1411, 1230, 1028, 742 $cm^{-1}$; MS-FAB *m/z* 840 $[M + H]^+$.

KAR-3 sulphate: m.p.> 300°C (decomposed); optical rotation (1% solution in water): $[\alpha_D]_0^{25}$ = +0.8°; $[\alpha_D]^{25}_{Hg\ 578}$ = +1.0°; $[\alpha_D]^{25}_{Hg\ 546}$ = +1.2°; $[\alpha_D]^{25}_{Hg\ 436}$ = +5.8°; $[\alpha_D]^{25}_{Hg\ 365}$ = +25.9°.

***Synthesis of KAR-4*** [3′-allyl-2′,4′-dioxo-3,5′-spiro-oxazolidino-4-deacetoxy-vinblastine] (Figure 1B; $R_1$, $CH_3$; $R_2$, $CH_2CH{=}CH_2$). The steps of synthesis of KAR-4 are the same as that of KAR-2, but allyl isocyanate (10 ml, 9.4 g, 113.25 mmol) was used instead of chloroethyl-isocyanate. The yield was 25% and 88% for KAR-4 and KAR-4 sulphate, respectively.

KAR-4: IR $\upsilon_{max}$ (potassium bromide): 3467, 1813, 1743, 1617, 1402, 1240, 1129, 1036, 741 $cm^{-1}$; MS-FAB *m/z* 804 $[M + H]^+$.

KAR-4 sulphate: m.p. > 300°C (decomposed); optical rotation (1% solution in water): $[\alpha_D]0^{25}$ = +1.1°; $[\alpha_D]^{25}_{Hg\ 578}$ = +1.4°; $[\alpha_D]^{25}_{Hg\ 546}$ = +1.8°; $[\alpha_D]^{25}_{Hg\ 436}$ = +7.3°; $[\alpha_D]^{25}_{Hg\ 365}$ = +30.8°.

### Analytical methods

Reaction progress and purity were monitored by analytical thin-layer chromatography on strips of Merck 3552 plastic-backed silicon dioxide. Developed strips were viewed under light of 254 and 365 nm wavelengths. NMR measurements were carried out on a Varian Unity plus-500 instrument (500 MHz for $^1H$ and 125 MHz for $^{13}C$) in DMSO. Chemical shifts were determined relative to dTMS = 0.00 p.p.m. $^1H$ and $^{13}C$ signal assignments were verified by a concerted use of two-dimensional $^1H$–$^1H$ and $^1H$–$^{13}C$ shift correlation methods (DQFCOSY, HSQC, HMBC) and NOESY experiments. The gradient-enhanced DQFCOSY, HSQC, HMBC and NOESY experiments were recorded by using the standard spectrometer software package. A mixing time of $t_{mix}$ = 0.5 s was used for the NOESY experiments. An IR spectrum was obtained from potassium bromide pellets on a Nicolet-20 DxC FT-IR spectrometer and was consistent with the assigned structure. Melting point (m.p.) was taken on an Electrothermal digital m.p. apparatus (IA 8103) and is uncorrected. Mass spectrum was recorded on a Kratos MS 80 instrument using FAB.

### In vivo anti-tumour effects in P388 sensitive and resistant leukaemias

Mouse leukaemia P388 cells were maintained on DBA/2 inbred mice and transplanted by the intraperitoneal (i.p.) route to groups consisting of six BDF1 hybrid mice each. A dose of $10^6$ tumour cells per animal each was administered (five or six mice per group). For antitumoral studies, the drugs were dissolved in physiological saline and the solutions were administeredd using single doses i.p. during the 24th h after transplantation. The body weight and condition of the animals were registered daily. The effect achieved on the treated animals, i.e. the anti-tumour activity, was expressed as percentage of the life span related to the average life span (in days) of the control group [T/C(%)]:

$$T/C(\%) = \frac{\text{median day of survival of treated animals} \times 100}{\text{median day of survival of control animals}}$$

### In vivo anti-tumour effects in Ehrlich ascites carcinoma

Mouse Ehrlich ascites tumour cells were maintained on C57BL/65co mice and transplanted by i.p. route to groups consisting of three C57BL/65co mice each. The animals were 8 weeks old, weighing over 30 g and they were housed in a climate-controlled pathogen-limited environment with standard rodent feed and clean water ad libitum. Animals before tumour implantation were selected using a random number chart without regard to treatment group assignment. A dose of $2 \times 10^7$-tumour cells per animal (100 µl) i.p. was injected. The cell concentration and viability were checked before and after the tumour administration. For anti-tumour studies, newly synthesized and reference drugs were dissolved in Dulbecco's phosphate-buffered saline (Sigma), and the solution was administered i.p. using a single dose 4 days after transplantation. The body weight and condition of the animals were registered daily. The animals were sacrificed 8 days after injection of tumour and 4 days after the administration of drugs. Tumour volume was measured using a calibrated syringe. Also, tumour cell number was counted using a Coulter Multisizer II AccuComp (Coulter Corporation) and flow cytometry with propidium iodide 18 µg $ml^{-1}$. Viability was also checked by the latter method. Propidium iodide excitation was obtained

 *© Cancer Research Campaign 1999*

with an argon ion laser adjusted to deliver 15 mW at 488 nm, and fluorescence was detected using a 675-nm band-pass filter.

### Microculture tetrazolium assay (MTA)

Human neuroblastoma cell line (SH-SY5Y) was cultured in 75 $cm^2$ flask with NCTC-135 medium (Sigma) + 1% antibiotic + 10% fetal bovine serum. Cells were trypsinized, counted by trypan blue exclusion and dispersed within replicate 24-well culture plates (1 ml per well and 75 000 cells $ml^{-1}$) in 100 μl volumes containing the same medium but only supplemented with 2% fetal bovine serum. After a 24 h incubation at 37°C, 5% carbon dioxide, 100% relative humidity in Heraeus Forma Scientific 3862 incubator, KAR derivatives were inoculated at various concentrations (control group, $n = 12$; each drug treatment group, $n = 4$). Medium/tetrazolium reagent blank ($n = 4$) was utilized for 'background' determination. Culture plates were then incubated at 37°C for 4 days before the addition of tetrazolium reagent. Forty microlitres of 50 mg $ml^{-1}$ 3-(4,5-dimethylthiazol-2-yl)-2,5-diphenyltetrazolium bromide (MTT) was added to each culture well (resulting in 0.2 mg $ml^{-1}$ final concentration) and cultures were incubated at 37°C for 24 h. MTT-formazan is generated by cellular reduction of the MTT tetrazolium reagent. Assuming that cellular reductive activity is constitutive and remains relatively constant throughout the duration of an experiment, the viable cell number is directly proportional to the production of formazan, which was measured after solubilization (Finlay et al, 1986). After incubation, cell monolayers and formazan were inspected microscopically. Then the culture medium supernatants were removed from wells by slow aspiration (needle suction) and replaced with 500 μl DMSO. After formazan solubilization (vibration on a plate shaker), the absorbance of each well was measured spectrophotometrically at 540 nm. Cell line growth and growth inhibition were expressed in terms of mean (± s.d.) absorbance units and/or percentage of control absorbance (± s.d.) after subtraction of mean 'background' absorbance.

### Electron microscopy

Samples for electron microscopy were prepared by incubating 10 μM tubulin, 20 μM taxol, 2 μM PFK without and with drugs (2 μM) for 30 min at 30°C. For negative staining, a drop from the unpelleted samples was applied to formvar/carbon-coated glow-discharged copper grids for 30 s. The solution was removed and the grid stained with one drop of freshly filtered 1% aqueous uranyl acetate for 30 s. The excess of stain was removed by blotting with filter paper. The specimens were examined and photographed in a Jeol CX 100 electron microscope operated at an accelerating voltage of 80 kV. Magnification was calibrated with a diffraction grating replica (2160 l $mm^{-1}$, Balzers).

### Turbidity measurements

Tubulin was incubated in the presence and absence of drugs at different concentrations in 50 mM MES buffer at pH 6.8 containing 100 mM, potassium chloride, 5 mM magnesium chloride, 2 mM dithioerythritol at 37°C. The drugs were added to the thermostated solution in the cuvette. The assembly was started with the addition of taxol to the samples in a final concentration of 20 μM from 10 μM stock solution in DMSO. Change of absorbance was monitored at 350 nm by a Hewlett Packard 8451A spectrophotometer. The effects of drugs were determined from the initial rates of the polymerization curves measured at different drug concentrations. The $IC_{50}$ values of the drugs were calculated from the dose–response curves with the method of maximum likelihood probit analysis.

### Pelleting experiments

Tubulin polymerization was carried out in 50 mM Hepes, 100 mM potassium chloride, 5 mM magnesium chloride at pH 7.0, at 30°C with 10 μM tubulin. For vinblastine or KAR-2-treated samples, tubulin was preincubated with 2 μM drug in the absence and presence of 2 μM PFK. The assembly was started by addition of taxol to the samples in a final concentration of 20 μM. Then the samples were centrifuged at 100 000 ***g*** for 20 min at 30°C. Under these conditions, microtubules and microtubule-bound PFK are completely pelleted, whereas non-microtubule-bound (uncomplexed) PFK and non-polymerized tubulin dimers remain in the supernatant (Lehotzky et al, 1993, 1994; Vértessy et al, 1996). The protein amount in the pellet and supernatant fractions was quantified by densitometry after SDS-PAGE separation in 9% polyacrylamide gels using the BioRad GelDoc 1000 densitometer with the Molecular Analyst software (Vértessy et al, 1996, 1997).

### Anti-calmodulin activity assay

PFK (6 mg $ml^{-1}$) was extensively dialysed against 0.1 M potassium phosphate buffer, pH 8.0, containing 5 mM dithioerythritol to remove ammonium sulphate. For preincubation of PFK with calmodulin and drugs, the enzyme was diluted to 1.2 μM protomer concentration into Hepes buffer containing 100 mM potassium chloride, 10 mM magnesium chloride, 10 mM disodium hydrogen phosphate and 100 μM calcium chloride. The final concentrations of calmodulin and drugs were 3 μM and 20 μM respectively. After 60 min, 10-μl aliquots were withdrawn to assay PFK activity in 1 ml at 37°C in 50 mM Tris, pH 8.0, containing 2 mM fructose-6-phosphate, 1 mM ATP, 3 mM magnesium chloride, 0.2 mM NADH, 3 mM dithioerythritol, 0.1 mM ethylene glycol bis(β-aminoethylether)-N,N,N′,N′-tetraacetic acid, 2 units of aldolase, 12 units of triosephosphate isomerase, and 2 units of glycerol-3-phosphate dehydrogenase (Mayr, 1987; Lehotzky et al, 1993). The reaction was followed by NADH consumption at 340 nm using Simadzu spectrophotometer. The specific activity of PFK was 159 U $mg^{-1}$ measured at 1 μg $ml^{-1}$ final concentration after dilution from 1 mg $ml^{-1}$ stock solution into activity assay.

### RESULTS

### Structural characterization of the synthesized KAR derivatives

KAR-2 was readily synthesized from 4-deacetoxy-vinblastine occurring in significant amounts in *Catharanthus roseus*. Substituents of the C3 atom of the vindoline moiety, $COOCH_3$ and OH, were cyclized into an oxazolidino ring using a bifunctional reagent, β-chloroethyl-isocyanate. The synthesis of KAR-4 was performed in a similar way using allyl isocyanate as reagent. For synthesis of KAR-3, 4-deacetoxy-vincristine was used which was obtained by oxidation of 4-deacetoxy-vinblastine using $n\text{-}Bu_4NMnO_4$ as oxidation agent. In this way, the yields were only

© Cancer Research Campaign 1999

**Table 1** Effect of drugs in various tests

| Drug | In vivo effect P388 (single doses) Maximal effect T/C (%[a]) | Dose (mg kg$^{-1}$) | Cytotoxicity MTA $IC_{50}$ (μM) | Anti-tubulin effect Turbidity at 350 nm[c] $IC_{50}$ (nM) | Anti-calmodulin activity Effect on calmodulin induced PFK inactivation[d] Activity (U mg$^{-1}$) | Efficiency (%) |
|---|---|---|---|---|---|---|
| KAR-2 | 207 ± 24[b] | 60[b] | 0.31 ± 0.06 | 200 ± 79 | 11.1 ± 0.6 | 5 |
| KAR-3 | 187 ± 40 | 20 | 0.31 ± 0.05 | 136 ± 61 | 34.4 ± 1.8 | 45 |
| KAR-4 | 214 ± 21 | 40 | 0.35 ± 0.01 | 185 ± 83 | 10.9 ± 0.7 | 5 |
| Vinblastine | – | – | 0.005 ± 0.001 | 248 ± 42 | – | 61[e] |
| Vincristine | 147 ± 36[b] | 1[b] | 0.013 ± 0.001 | 290 ± 79 | – | 62[e] |
| Trifluoperazine | – | – | – | – | 46.1 ± 2.2 | 65 |

For experimental details see Materials and methods.

[a] $T/C\ (\%) = \frac{\text{median day of survival of treated animals}}{\text{median day of survival of control animals}} \times 100$

Number of animals used: six per groups

[b] Data published also in Orosz et al (1997a). [c] Polymerization curves of tubulin were obtained by measuring turbidity (absorbance at 350 nm) in the absence and presence of drugs. Tubulin was used at 20 μM concentration. The data are the average of three independent measurements. [d] Concentrations of PFK (in protomers), calmodulin and drugs were 1.2 μM, 3 μM and 20 μM respectively. Residual activity of PFK after 60 min incubation in the absence and presence of calmodulin was 66.6 U mg$^{-1}$ (100%) and 8.0 U mg$^{-1}$ (0%) respectively. Efficiency was calculated from the activity data by the following formula:

$$\text{Efficiency} = \frac{\text{Activity}_{\text{PFK–CaM–drug}} - \text{Activity}_{\text{PFK–CaM}}}{\text{Activity}_{\text{PFK}} - \text{Activity}_{\text{PFK–CaM}}}$$

[e] Data taken from Molnár et al (1995).

25–30% because of the side reactions, however the reactive chloroethyl or allyl groups may serve as initial points of further modifications. KAR-2, KAR-3 and KAR-4 were separated from byproducts by silica gel chromatography. The sulphate salts were prepared from the drugs dissolved in ethanol and sulphuric acid.

The structures of all the three KAR derivatives were verified by high-field NMR measurements in the liquid phase. $^{1}$H-NMR and $^{13}$C-NMR data are consistent with the structures of Figure 1*. We note that all three bis-indols exhibit complex internal dynamics. In particular, the flexibility of the nine-member ring gives much motional degree of freedom to the system. In addition, rotation about the C(18′)–C(15) bond gives further conformational species. In each molecule, the NMR spectra exhibit conspicuously broad resonances, which indicates that at least one of these internal motions is moderately slow on the chemical shift time scale. The pronounced exchange broadening persisted even at elevated temperatures (45°C for KAR-2 and at 49°C for KAR-3 and KAR-4), which led to a masking of the $^{1}$H–$^{1}$H coupling patterns and the extreme widening of some C–H proton and carbon resonances. For example, the proton signals due to C(3)$H_2$, C(5)$H_2$ and C(21)H could only be identified through the 2D $^{13}$C–$^{1}$H correlation experiments. Nevertheless, the combined use of COSY, HSQC and HMBC and NOESY measurements proved to be successful in establishing the $^{1}$H–$^{1}$H and $^{1}$H–$^{13}$C scalar and dipolar networks in the molecules.

*$^{1}$H and $^{13}$C chemical shift data for the sulphuric acid salts of compounds KAR-2, KAR-3 and KAR-4 will be published elsewhere.

### In vivo anti-tumour potency on P388 mouse ascites leukaemia

Previously, we demonstrated that KAR-2 displays significant anti-tumour potency on P388 mouse leukaemia (Orosz et al, 1997*a*). Both KAR-3 and KAR-4 have a very similar dose-dependent effect on this tumour to KAR-2. Based on the possibility of administering high doses as observed during the experiments, the tests were carried out by using single doses. This administrational schedule revealed that KAR derivatives were effective in a single i.p. dose of 20 mg kg$^{-1}$. Their maximal effects were about 200 T/C(%). This value was reached using 20 mg kg$^{-1}$, 40 mg kg$^{-1}$ and 60 mg kg$^{-1}$ for KAR-3, KAR-4 and KAR-2 respectively (Table 1). The maximal tolerated dose were extremely high for KAR-2 (80 mg kg$^{-1}$), even in comparison to the other KAR derivatives.

### In vivo anti-tumour potency on Ehrlich ascites carcinoma

A very important factor for testing the anti-tumour ability of a novel compound is its preserved activity when treatment is delayed after the graft, i.e. a preserved efficiency of this drug when the tumour is sufficiently expanded which better mimicked the clinical reality. For this purpose, KAR derivatives (40 mg kg$^{-1}$) were applied for treatments to compare the anti-tumour potencies of the new compounds in the Ehrlich carcinoma test. As shown in Table 2A, each of the KAR derivatives display a significant effect on the tumour cell proliferation, however KAR-2 and KAR-3 seem to be more effective in decreasing the cell number than

*© Cancer Research Campaign 1999*

**Table 2** In vivo antitumour effects on Ehrlich ascites carcinoma after treatment with single dose of drugs

**Table A**

| Drug | Dose ($mg\ kg^{-1}$) | Volume of tumour per mouse (ml) | Cells $ml^{-1}$ (per cent of control)[a] | Total cells (per cent of control)[a] |
|---|---|---|---|---|
| Initial value[b] | | 1.33 | 10 ± 3 | 3.2 ± 3 |
| Control | | 5.33 | 100 ± 4 | 100 ± 4 |
| KAR-2 | 40 | 2.33 | 56 ± 6 | 24 ± 6 |
| KAR-3 | 40 | 3.00 | 57 ± 8 | 32 ± 8 |
| KAR-4 | 40 | 3.33 | 90 ± 14 | 57 ± 14 |

**Table B**

| Drug | Dose ($mg\ kg^{-1}$) | Volume of tumour per mouse (ml) | Cells $ml^{-1}$ (per cent of control)[a] | Total cells (per cent of control)[a] |
|---|---|---|---|---|
| Control | | 2.67 | 100 ± 2 | 100 ± 2 |
| Vincristine | 1 | 2.00 | 157 ± 2 | 57 ± 2 |
| | 2 | 1.50 | 106 ± 2 | 60 ± 2 |
| | 5 | 0.64 | 225 ± 2 | 54 ± 2 |
| | 10 | 0.53 | 224 ± 2 | 45 ± 2 |
| | 15[c] | – | – | – |
| KAR-2 | 20 | 1.97 | 63 ± 2 | 46 ± 2 |
| | 40 | 1.17 | 60 ± 6 | 24 ± 6 |
| | 60 | 1.70 | 46 ± 2 | 29 ± 2 |
| | 80[d] | 0.45 | 211 ± 2 | 36 ± 2 |

Mouse Ehrlich ascites tumour cells were maintained on C57BL/65co mice and transplanted by i.p. route. Drugs were administered i.p. 4 days after tumour implantation. The animals (three per group) were sacrificed 8 days after implantation of the tumour (4 days after administration of drugs). For other details see Materials and methods.
[a]Tumour cell numbers from treated mice are expressed as percentage of the control:tumour-treated mice without drug administration sacrificed 8 days after implantation of the tumour.
A: 100% equals 1240 ±4% × $10^6$ cells $ml^{-1}$ and 6769±4% × $10^6$ total cell number.
B: 100% equals 275 ±1.4% × $10^6$ cells $ml^{-1}$ and 734±1.4% × $10^6$ total cell number.
[b]Determined 4 days after tumour implantation, before the administration of the drugs. [c]The animals died before the sacrifice.[d]The animals were dying in the same day of the sacrifice.

KAR-4. Because KAR-3 showed lower T/C(%) at single dose administration in P388 test (Table 1) than KAR-2, the latter compound was further investigated and its efficiency was compared with that of the clinically relevant vincristine in a dose-dependent way (Table 2B). The KAR-2 and vincristine doses used for treatments were varied in different ranges to be effective but not toxic. KAR-2 and vincristine inhibited tumour cell proliferation in all the doses used. Significant decrease of the tumour cell density can be observed in the cases of both drug treatments as a function of concentration. As shown in Table 2B, the total cell numbers reached the lowest values when the animals were treated with 40 mg KAR-2 $kg^{-1}$ mouse (24% of the control). In contrast, the maximum dose of vincristine that did not result in the death of the animals (10 mg vincristine $kg^{-1}$ mouse) decreased the total cell number to only 45% of the control value. The cell viability appeared to be well maintained (over 98%) after the treatments. The treated animals showed consistent body weight patterns as well as eating and drinking habits with the controls during the experiments. This finding shows that at the selected doses the treatments were well tolerated by the mice, except at the highest dose of vincristine which caused the death of two of the three treated animals. It can be, therefore, concluded from these in vivo experiments that KAR-2 is more effective in inhibiting tumour cell proliferation than vincristine.

### Cytotoxicity of KAR derivatives in neuroblastoma cells

The cytotoxicity on human neuronal tumour cells caused by KAR derivatives was quantified by a MTA involving DMSO solubilization of MTT-formazan (Alley et al, 1988). We used this approach to discriminate between viable and unviable cell groups in KAR derivative-treated neuroblastoma cell culture. The drugs were added to neuroblastoma cells in various concentrations, and after 4-day treatments the viable cells were quantified by MTA. As shown in Table 1, the growth inhibitory concentration range observed with KAR derivatives was about 0.3 μM $IC_{50}$. This value was significantly lower for vincristine and vinblastine ($IC_{50}$ = 13 nM and 5 nM, respectively).

© Cancer Research Campaign 1999

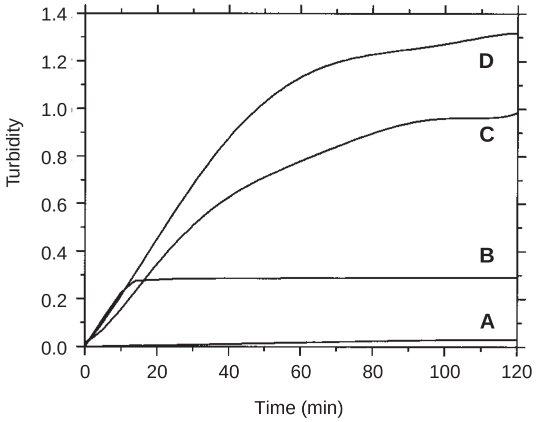

**Figure 2** Polymerization curves of 10 μM tubulin obtained by measuring turbidity in the absence (A) and (B) and presence (C) and (D) of 2 μM PFK. Tubulin was polymerized under conditions as described in Materials and methods. Turbidity was measured at 350 nm in the absence (B) and (D) and in the presence (A) and (C) of 2 μM KAR-2

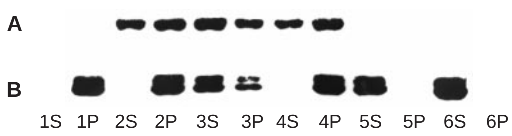

**Figure 3** Dependence of drug effects on microtubule ultrastructure: SDS-PAGE of drug-treated samples. Single and bundled microtubules were prepared in the absence and presence of PFK, respectively, as described in Materials and methods. Supernatants (S) and pellets (P) of untreated and vinblastine- or KAR-2-treated samples were assayed in SDS-PAGE. Samples are: 1, control; 2, plus PFK; 3, plus PFK and vinblastine; 4, plus PFK and KAR-2; 5, plus vinblastine; 6, plus KAR-2. (**A**) PFK; (**B**) tubulin α and β subunits

### Antimicrotubular activity

Because KAR derivatives were found to be effective in antitumour tests in vivo, we compared their antimicrotubular activities in vitro in a tubulin assembly system. The polymerization was induced by taxol and visualized by turbidimetry (Schiff et al, 1979). The half-maximum inhibitions ($IC_{50}$) occurred between 100 and 200 nM for KAR derivatives and between 250 and 300 nM for vinblastine and vincristine (Table 1). The inhibitory effect of KAR-3 is slightly higher than that of the other two KAR derivatives.

### Selectivity of antimicrotubular activity

To assess whether the effect of the KAR derivatives depends on the organization state of the tubulin/microtubule system, the PFK-induced bundling (cross-linking) process was analysed in the presence and absence of the drugs. The bundling activity of PFK was previously demonstrated in vitro (Lehotzky et al, 1994). The concentration of drugs (2 μM) was selected to inhibit significantly the tubulin polymerization in the absence of PFK (cf. the $IC_{50}$ values in Table 1) (Figure 2, curves A and B). When tubulin assembly was monitored by turbidity measurements in the presence of KAR-2 and PFK, we observed that the turbidity is extensively increased, suggesting that PFK can overcome the inhibitory effect of KAR-2 on tubulin polymerization (Figure 2, curves C and D). The effects of KAR-3 and KAR-4 on tubulin polymerization were similar to that of KAR-2, both in the absence and presence of PFK (data not shown). These results are similar to those obtained with vinblastine (Lehotzky et al, 1994), however the resistance of bundled microtubules was higher when KAR-derivatives were used.

The results of pelleting experiments are consistent with the turbidity measurements. To detect the alteration in the microtubule formation caused by the drugs, the centrifugation speed was chosen such that single and PFK-bundled microtubules were pelleted while tubulin dimers or small oligomers were in the supernatant. Therefore, the partition of tubulin in the pellets and the supernatants is indicative to the effect of drugs expressed on the single and bundled microtubules. Figure 3 shows the gel electrophoresis pictures of the pellets and supernatants of the untreated, KAR-2- or vinblastine-treated tubulin samples. The double bands are characteristic of the α and β subunits of tubulin dimers. In the absence of drugs with and without PFK, tubulin can be visualized only in the pellet, indicating that all tubulins were assembled into the microtubule under the experimental conditions used. In the PFK-containing sample, an additional band appears, the position of which corresponds to the molecular mass of the PFK subunit. If the assembly was carried out in the presence of drugs but without PFK, practically all tubulin appears in the supernatants showing that both drugs prevented the microtubule formation. When the tubulin polymerization was carried out with PFK, significant amount of tubulins were assembled into microtubules

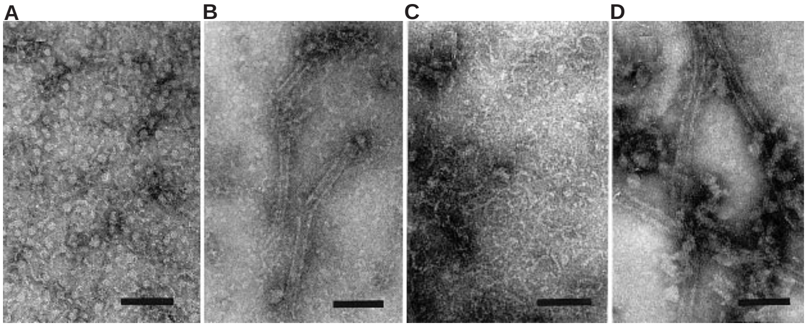

**Figure 4** Electron micrographs of tubulin samples containing vinblastine (**A**), vinblastine plus PFK (**B**), KAR-2 (**C**), and KAR-2 plus PFK (**D**) prepared and stained as described in the Materials and methods. Microtubules are present in **B** and **D**; they are, however, absent in **A** and **C**. All of the samples contain numerous C-shaped and thread-like oligomers. Bars, 60 nm

© Cancer Research Campaign 1999

even in the presence of vinblastine, and virtually no depolymerizing effect of KAR-2 can be observed. This finding provides evidence that the antimicrotubular activity of KAR-2 is highly dependent on the ultrastructure of microtubules.

Electron microscopic examination of the tubulin polymer formed in the simultaneous presence of drugs (2 μM) and PFK (2 μM) supports these conclusions. As shown in Figure 4, numerous short microtubules are seen in samples containing tubulin-PFK and drug (vinblastine or KAR-2), however they are absent in samples prepared without addition of PFK. Similar results were obtained with KAR-3 and KAR-4 (data not shown). A common feature of the samples is that they contain many C-shaped aggregates that probably represent tubulin ligomers. However, no such profiles were seen in samples assembled in the presence of PFK without addition of drugs (Lehotzky et al, 1994).

### Anti-calmodulin activities

$Ca^{2+}$-calmodulin-modulated PFK activity is a well-established system to test anti-calmodulin activity of drugs (Orosz et al, 1988, 1990). The affinity of drugs and PFK to calmodulin is comparable, which renders it possible to use relatively low drug concentrations and thus the direct binding of drugs to PFK can be disregarded (Orosz et al, 1988). In addition, our previous data suggested that the inhibitory potency of drugs suppressing the modulating effect of calmodulin manifested itself in both phosphodiesterase and PFK systems in a similar manner (Orosz et al, 1990).

To compare the anti-calmodulin activities of KAR derivatives, KAR-2, KAR-3 and KAR-4, their effects on the calmodulin-stimulated inactivation of PFK was measured. The experimental system was adjusted to get, on one hand, a high degree of inhibition of PFK by calmodulin, and on the other hand a significant amount of calmodulin to be complexed with PFK. PFK (1.2 μM) in protomers was preincubated with and without $Ca^{2+}$-calmodulin in the absence and presence of drugs. KAR derivatives as well as trifluoperazine, a classic calmodulin antagonist, as a reference molecule were added at 20 μM final concentrations. After 60 min incubation, the samples were assayed for PFK activity. The results are summarized in Table 1. According to expectation (Ovádi, 1989), the inhibitory effect of calmodulin on PFK activity was significantly attenuated by trifluoperazine, but not at all by KAR-2 (Orosz et al, 1997*b*). As shown in Table 1, KAR-4, similar to KAR-2 (both are vinblastine derivatives), does not display anti-calmodulin activity in the PFK assay, whereas KAR-3 can extensively reduce the calmodulin-mediated inactivation of PFK by about 50% suggesting its significant anti-calmodulin potency.

### DISCUSSION

Bis-indol derivatives are among the most potent antimitotic agents expressing their activities predominantly on tubulin/microtubule system. Active derivatives, such as vinblastine and vincristine, are isolated from plant extract. These drugs are extensively used in chemotherapeutic treatments and they are very effective to arrest mitosis of cells, however there are limitations in their clinical administrations. One of the problems is the limited amount of these drugs extracted from *Catharanthus roseus*. There are strategies to produce new potent molecules by derivation of natural molecules. We have synthesized novel compounds, KAR-2, KAR-3 and KAR-4, which are semisynthetic derivatives of bis-indols occurring in the *Catharanthus roseus* extract in relatively large amounts. The KAR derivatives were synthesized from 4-deacetoxy-vinblastine or 4-deacetoxy-vincristine. Substituents of the C3 atom of the vindoline moiety, $COOCH_3$ and OH, were cyclized into oxazolidino ring using bifunctional reagents (Figure 1). The synthesis is a relatively simple process and it can not be fulfilled by good efficiency (yields are between 25 and 30%), however the reactive chloroethyl or allyl groups may serve as initial points of further modifications.

These newly synthesized compounds differ chemically from vinblastine and vincristine by the substitution of the 3 and 4 carbon atoms of the vindoline moiety: the acetoxy group on the C4 atom is replaced by H atom and it contains differently substituted oxazolidino group on the C3 atom. Oxazolidino derivatives of bis-indol alkaloids substituted by acetoxy and acetyl groups at position C4 were synthesized by Eli Lilly (Miller and Antowsk, 1984) as well. However, those compounds investigated in binding assays in vitro (Codaccioni et al, 1988) and on different tumours (Lathan et al, 1985) were not found to be superior to vinblastine or vincristine.

Turbidity measurements revealed that the KAR derivatives displayed significant inhibitory effects on tubulin polymerization in vitro. Obviously, the inhibition by drugs at a given drug concentration is highly dependent on the system used as polymerization assay. For example, in Hepes and glutamate systems, the inhibitory effects of antimitotic drugs manifested themselves at lower and higher drug concentrations, respectively, than in MES buffer (Orosz et al, 1997*a*). Nevertheless, the tendency that KAR derivatives, especially KAR-3, inhibit in vitro tubulin assembly at lower drug concentrations than vinblastine and vincristine is clear.

In contrast, we found that if the polymerization was carried out in the presence of a crosslinking protein, PFK, the microtubule formation was much less inhibited by KAR derivatives than in the absence of the bundling protein. This observation suggests that the effect of bis-indols, including KAR-derivatives, on microtubule assembly depends highly on the ultrastructural state of microtubules, and the structural changes of KAR derivatives does not cause a quantitative difference in this effect.

This exciting phenomenon may possess pharmacological relevance. Depending on the cell type and cellular conditions, the relative amounts of tubulin dimers, microtubules and bundled microtubules vary in a wide range. For example, bundling of microtubules can be induced by MAPs and other proteins (e.g. PFK), or the ATP level modulates the enzyme-induced bundling of tubules (Durrieu et al, 1987; Lehotzky et al, 1994). Our data suggest that the bundled microtubules become resistant, at least partially, to these antimitotic agents. In contrast, rapidly growing tumour cells are characterized by a high rate of glycolysis with a concomitantly high ATP level. Several mechanisms are responsible for this property (Schneider, 1981). As we suggested recently (Vértessy et al, 1997), one of the possibilities is that PFK is not attached to microtubules at a relatively high MgATP level (Lehotzky et al, 1994; Vértessy et al, 1997), therefore, in its unbound form, it exhibits high overall activity ensuring high glycolytic flux. At MgATP levels high enough to prevent PFK binding to microtubules, kinase-induced cross-bridging of tubules cannot occur, possibly rendering single tubules more sensitive to bis-indol treatment than cross-bridged microtubules.

Mouse leukaemia P388, an in vivo tumour model, was suggested as a reasonable prescreen because it is sensitive to most classes of

© Cancer Research Campaign 1999

clinically effective drugs, but is sufficiently restrictive to avoid overloading the panel (Cros et al, 1989; and references therein). Our previous data showed that KAR-2 is an effective drug in this test and its efficacy is similar to or slightly lower than those of vincristine and vinblastine in repeat doses, although its effective dose is higher. T/C values were 199% and 174% for vinblastine (0.2 mg $kg^{-1}$) and KAR-2 (1 mg $kg^{-1}$), respectively, at eightfold treatment (Orosz et al, 1997*a*). KAR-3 and KAR-4 behave similarly to KAR-2 (data not shown). Although the effective doses of KAR derivatives are higher than those of vinblastine/vincristine, the disadvantage of the vinblastine/vincristine administration in the chemotherapeutic treatments is that the prolonged treatments are frequently not effective because of the multidrug resistance problem. The administrational schedule revealed an advantageous property of the KAR derivatives: they were active even in a single high dose (Table 2), whereas single doses of vincristine were less effective or caused the death of the animals. A similar observation was noted in another type of experiment using mice hosting Ehrlich ascites tumour cells, in which tumour was allowed to develop for 4 days before drug administration. The fact that significant prolongation of life span could be reached by a single high, but not toxic, dose of the KAR derivatives makes them potential candidates for further pharmacological and clinical investigations. However, it remains to be tested whether these drugs have the same characteristics in the clinical treatment. Additionally, we have observed in experiments with a neuroblastoma cell line that KAR derivatives exhibit similar cytotoxicity, but their $IC_{50}$ values are significantly higher than that of vincristine in spite of the fact that their antimicrotubular activities are in the same order of magnitude.

Calmodulin as a key calcium receptor protein modulates several very different cellular functions and metabolism. Previously, we have reported that vinblastine, vincristine and KAR-2 interact with calmodulin, their affinities are comparable with that of tubulin and that their interaction is a $Ca^{2+}$ dependent process (Molnár et al, 1995; Orosz et al, 1997*b*). However, we found that KAR-2 does not display anti-calmodulin activity in the PFK assay (Orosz et al, 1997*b*). To investigate whether the lack of anti-calmodulin potency resides only on the spiro-oxazolidino portion of KAR-2, we tested the other KAR derivatives for anti-calmodulin potency. KAR-2 and KAR-4 do not have calmodulin antagonist effect in a calmodulin-modulated enzymatic test, whereas KAR-3 can partially suspend the modulating effect of calmodulin. Therefore, we conclude that the ‘extra’ ring of these compounds is responsible for the decreased or diminished anti-calmodulin activities; in contrast, the substitution of the methyl group (KAR-2, KAR-4) by a formyl one (KAR-3) at a position apart from the oxazolidino ring can selectively modify the anti-calmodulin potency of KAR derivatives. Although we do not have unambiguous evidence for the direct relationship between anti-calmodulin potency and undesired toxic side-effects of bis-indols, our data allow one to speculate about the relationship of these effects.

### ABBREVIATIONS

MES, 2-(N-morpholino)-ethanesulphonic acid; MTA, microculture tetrazolium assay; MTT, 3-(4,5-dimethylthiazol-2-yl)-2,5-diphenyltetrazolium bromide; PFK, phosphofructokinase [ATP: D-fructose 6-phosphate 1-phospho-transferase].

### ACKNOWLEDGEMENTS

This work was supported by grants from the Hungarian National Science Foundation OTKA (T-17830 and T-025291 to OJ and T-2227 to JK), the Hungarian Ministry for Education and Culture (TKFP 0158/97, 1023/97), the Hungarian National Committee for Technological Development, OMFB (E-28/97), by a Spanish government grant from DGICYT (PM 96-0099) and the European Community (Grant ERBIC15CT960307). JO thanks Catalan Autonomous Government (CIRIT) for supporting her visiting full professor position at University of Barcelona. We wish to thank Dr Zsuzsa Somfai who performed the P388 experiments.

### REFERENCES

Alley MA, Scudiero DA, Monks A, Hursey ML, Czerwinski MJ, Fine DL, Abbott BJ, Mayo JG, Shoemaker RH and Boyd MR (1988) Feasibility of drug screening with panels of human tumor cell lines using a microculture tetrazolium assay. *Cancer Res* **48**: 589–601

Codaccioni F, Dell’Amico M, Boudeaux M, Briand C and Lux B (1988) Influence of the guanine nucleotide phosphorylation state and of $Mg^{2+}$ ions on the interaction of vinzolidine/tubulin 6S. *Arch Biochem Biophys* **267**: 236–244

Cros S, Wright M, Morimoto M, Lataste H, Couzinier JP and Krikorian A (1989) Experimental antitumor activity of navelbine. *Semin Oncol* **16** (suppl. 4): 15–20

De Brabander M, Nuydens R, Geuens G, Moeremans M, De Mey J and Hopkins C (1980) Nanovid ultramicroscopy: a new non-destructive approach providing new insights in subcellular motility. In *Microtubules and Microtubule Inhibitors*, De Brabander M and De Mey J (eds), pp. 187–196. Elsevier: Amsterdam

De Bruyn A, De Taeye L, Simonds R, Verzele M and De Pauw C (1982) Alkaloids from *Catharanthus roseus*. Isolation and identification of 17-desacetoxyvinblastine and 17-desacetoxyleurosine. *Bull Soc Chim Belg* **91**: 75–85

Durrieu C, Bernier-Valentin F and Rousset B (1987) Binding of glyceraldehyde-3-phosphate dehydrogenase to microtubules. *Mol Cell Biochem* **74**: 55–65

Dustin P (1984) *Microtubules*. Springer-Verlag: Berlin

Finlay GJ, Wilson WR and Baguley BC (1986) Comparison of *in vitro* activity of cytotoxic drugs towards human carcinoma and leukaemia cell lines. *Eur J Cancer Clin Oncol* **22**: 655–662

Gopalakrishna R and Anderson WB (1982) $Ca^{2+}$-induced hydrophobic site on calmodulin: application for purification of calmodulin by phenyl-sepharose affinity chromatography. *Biochem Biophys Res Commun* **104**: 830–836

Hesterberg LK and Lee JC (1982) Self-association of rabbit muscle phosphofructokinase: effects of ligands. *Biochemistry* **21**: 216–222

Laemmli UK (1970) Cleavage of structural proteins during assembly of the head of bacteriophage T4. *Nature* **227**: 680–688

Lathan B, Clark GM and Von Hoff DD (1985) In vitro comparison of vinzolidine and vinblastine: a model for methods of evaluation of analogues in a human tumor cloning system. *Cancer Res* **45**: 6286–6289

Lehotzky A, Telegdi M, Liliom K and Ovádi J (1993) Interaction of phosphofructokinase with tubulin and microtubule: quantitative evaluation of the mutual effects. *J Biol Chem* **268**: 10888–10894

Lehotzky A, Pálfia Z, Kovács J, Molnár A and Ovádi J (1994) Ligand-modulated cross-bridging of microtubules by phosphofructokinase. *Biochem Biophys Res Commun* **204**: 585–591

Liliom K, Lehotzky A, Molnár A and Ovádi J (1995) Characterization of tubulin–drug interactions by ELISA. *Anal Biochem* **228**: 18–27

Lin CM, Singh SB, Chu PS, Dempcy RO, Schmidt JM, Pettit GR and Hamel E (1988) Interactions of tubulin with potent natural and synthetic analogs of the antimitotic agent combretastin: a structure-activity study. *Mol Pharmacol* **34**: 200–208

Mayr GW (1987) Interaction of calmodulin with phosphofructokinase: binding studies and evaluation of enzymatic and physicochemical changes. *Methods Enzymol* **139**: 745–763

Miller JC and Antowsk GE (1984) BE 861 417. *Drugs Future* **9**: 913

Molnár A, Liliom K, Orosz F, Vértessy BG and Ovádi J (1995) Anti-calmodulin potency of indol alkaloids in *in vitro* systems. *Eur J Pharmacol* **291**: 73–82

Na CN and Timasheff SN (1986) Interaction of vinblastine with calf brain tubulin: multiple equilibria. *Biochemistry* **25**: 6214–6222

 *© Cancer Research Campaign 1999*

Orosz F, Christova TY and Ovádi J (1988) Functional *in vitro* test of calmodulin antagonism: effect of drugs on interaction between calmodulin and glycolytic enzymes. *Mol Pharmacol* **33**: 678–682
Orosz F, Telegdi M, Liliom K, Solti M, Korbonits D and Ovádi J (1990) Dissimilar mechanisms of action of anti-calmodulin drugs: quantitative analysis. *Mol Pharmacol* **38**: 910–916
Orosz F, Kovács J, Löw P, Vértessy BG, Urbányi Z, Ács T, Keve T and Ovádi J (1997*a*) Interaction of a new bis-indol derivative, KAR-2 with tubulin and its antimitotic activity. *Br J Pharmacol* **21**: 947–954
Orosz F, Vértessy BG, Salerno C, Crifo C, Capuozzo E and Ovádi J (1997*b*) The interaction of a new anti-tumor drug, KAR-2 with calmodulin. *Br J Pharmacol* **21**: 955–962
Ovádi J (1989) Effects of drugs on calmodulin-mediated enzymatic actions. A review. *Prog Drug Res* **33**: 353–395
Schiff PB, Fant J and Horwitz SB (1979) Promotion of microtubule assembly *in vitro* by taxol. *Nature* **277**: 665–667
Schneider F (1981) The aerobic glycolysis of tumor cells. *Naturwissenschaften* **68**: 20–27
Solti M, Dévay P, Kiss I, Londesborough J and Friedrich P (1983) Cyclic nucleotide phosphodiesterases in larval and brain of wild type and dunce mutant strains of *Drosophila melanogaster*: isozyme pattern and activation by $Ca^{2+}$/calmodulin. *Biochem Biophys Res Commun* **111**: 652–658
Vértessy BG, Kovács J and Ovádi J (1996) Specific characteristics of phosphofructokinase–microtubule interaction. *FEBS Lett* **379**: 191–195
Vértessy BG, Kovács J, Löw P, Lehotzky A, Molnár A, Orosz F and Ovádi J (1997) Characterization of microtubule-phosphofructokinase complex: specific effects of MgATP and vinblastine. *Biochemistry* **36**: 2051–2062
Watterson DM, Harrelson WG, Keller PM, Sharief F and Vanaman TC (1976) Structural similarities between the $Ca^{2+}$-dependent regulatory proteins of 3′:5′-cyclic nucleotide phosphodiesterase and actomyosin ATPase. *J Biol Chem* **251**: 4501–4513

© Cancer Research Campaign 1999 
